# Preliminary assessment of cavity‐nesting Hymenopterans in a low‐intensity agricultural landscape in Transylvania

**DOI:** 10.1002/ece3.7956

**Published:** 2021-08-01

**Authors:** Károly Lajos, Imre Demeter, Róbert Mák, Adalbert Balog, Miklós Sárospataki

**Affiliations:** ^1^ Department of Zoology and Ecology Hungarian University of Agriculture and Life Sciences Gödöllő Hungary; ^2^ Department of Horticulture Faculty of Technical and Human Science Sapientia Hungarian University of Transylvania Tirgu‐Mures Romania

**Keywords:** landscape context, solitary bees, solitary wasps, spider prey, spider‐hunting wasps, trap nests

## Abstract

In this study, our aim was to assess several traits of cavity‐nesting Hymenopteran taxa in a low‐intensity agricultural landscape in Transylvania. The study took place between May and August 2018 at eight study sites in the hilly mountainous central part of Romania, where the majority of the landscape is used for extensive farming or forestry. During the processing of the trap nest material, we recorded several traits regarding the nests of different cavity‐nesting Hymenopteran taxa and the spider prey found inside the nests of the spider‐hunting representatives of these taxa. We also evaluated the relationship between the edge density and proportion of low‐intensity agricultural areas surrounding the study sites and some of these traits.

The majority of nests were built by the solitary wasp genus *Trypoxylon*, followed by the solitary wasp taxa *Dipogon* and Eumeninae. Solitary bees were much less common, with *Hylaeus* being the most abundant genus. In the nests of *Trypoxylon*, we mostly found spider prey from the family of Araneidae, followed by specimens from the families of Linyphiidae and Theridiidae. In the nests of *Dipogon*, we predominantly encountered spider prey from the family of Thomisidae. We found significant effects of low‐intensity agricultural areas for the genera of *Auplopus*, *Megachile, Osmia*, and the Thomisid prey of *Dipogon*. We also found that the spider prey of *Trypoxylon* was significantly more diverse at study sites with higher proportions of low‐intensity agricultural areas.

Our results indicate that solitary bees seem to be more abundant in areas, where the influence of human activities is stronger, while solitary wasps seem to rather avoid these areas. Therefore, we suggest that future studies not only should put more effort into sampling in low‐intensity agricultural landscapes but also focus more on solitary wasp taxa, when sampling such an area.

## Graphical abstract

Nowadays, most regions of the European Union face an increasing agricultural intensification and urbanization, which partially caused a dramatic decline of insect diversity in the last few decades. However, even in the European Union, there are still a few regions and areas remaining, which are not under such a strong human influence and still harbor a considerably high insect diversity, like our study area, located in the central part of Romania. In our study, we found several new aspects concerning the diversity and distribution of solitary bees, wasps, and the spider‐hunting wasps' prey and also the effects of landscape context on these groups.

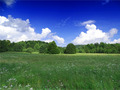

## 1 INTRODUCTION

Several recent studies have reported a decline of insect abundance, biomass, and species richness in many densely populated regions of Western Europe and also other parts of the world (Forister et al., 2019; Habel et al., 2019; Hallmann et al., 2017, 2021; Sánchez‐Bayo & Wyckhuys, 2019, 2021). (Wagner et al., 2021) The two main drivers behind this decline are the increasing agricultural expansion and intensification as well as urbanization in these regions, which lead to a loss or fragmentation of the insects' habitats (Habel et al., 2019; Knop, 2016; Merckx & Van Dyck, 2019; Piano et al., 2020; Raven & Wagner, 2021; Sánchez‐Bayo & Wyckhuys, 2019, 2021; Wagner, 2020). However, some other recent studies reported a partial recovery of insect abundance, biomass, and species richness in certain Western European regions (like in the Netherlands or Great Britain) since the 1990s, where different kinds of management actions or policies (e.g., stricter regulations of pesticide use, agri‐environmental schemes, conservation programs) have been implemented to protect and maintain (insect) biodiversity (Carvalheiro et al., 2013; Ollerton et al., 2014).

It has already been demonstrated in numerous studies that trap nests are useful tools to assess the biodiversity of cavity‐nesting Hymenopterans and also their trophic interactions in a certain area as well as the parasitoids and hyperparasitoids of these Hymenopteran taxa (Albrecht et al., 2007; Klein et al., 2006; Kruess & Tscharntke, 2002; Mayr et al., 2020; Scherber et al., 2010; Staab et al., 2018; Stangler et al., 2015; Steckel et al., 2014; Tscharntke et al., 1998). Basically, cavity‐nesting aculeate Hymenopterans can be divided into two trophic groups of nectar and pollen‐feeding solitary bees and predatory solitary wasps (Klein et al., 2006; Mayr et al., 2020; Steckel et al., 2014). With regard to the pollination service provided by cavity‐nesting solitary bees, which are pollinators of many wild and crop plant species, and the biological pest control by some cavity‐nesting solitary wasp species (like *Ancistrocerus gazella*; Harris, 1994), additional knowledge about these species and the influence of landscape context on them may provide help in measures for their protection.

The fact that trap nests provide a good nesting opportunity and thus lead to an accumulation of cavity‐nesting solitary Hymenopteran species living in the area surrounding these nests also makes trap nests especially suitable to study landscape effects. Some studies dealing with the effects of landscape context on cavity‐nesting Hymenopterans conducted rather simple landscape analyses looking only at the presence of (Holzschuh et al., 2009; Mayr et al., 2020; Tscharntke et al., 1998) or distance from certain habitat types like forests (Klein et al., 2006) or ecological compensation area (ECA) meadows (Albrecht et al., 2007). Other studies, however, looked more specifically at the landscape structure surrounding their study sites, analyzing the effects of the proportion of different habitat types (Coudrain et al., 2016; Kratschmer et al., 2020; Taki et al., 2008) or even conducting complex landscape analyses (Holzschuh et al., 2010; Steckel et al., 2014) at multiple spatial scales (Steckel et al., 2014; Taki et al., 2008).

Most previous studies, which were assessing cavity‐nesting Hymenopterans in different Western European countries, were conducted in high‐intensity agricultural landscapes (Table 1). However, in the eastern part of Europe, there are still a few regions and areas remaining, which are not under such a strong human influence. An example for such a region is Transylvania in the central part of Romania, where the population density is relatively low and the majority of the landscape is used for extensive farming or forestry. The most common form of extensive farming in this region is traditional small‐scale farming, which is characterized by manual hay mowing, manual hay gathering, and extensive low‐intensity organic manuring (Babai & Molnár, 2014; Babai et al., 2015). Such small‐scale pastures and meadows often harbor a high species diversity of insects and are regarded as high nature value (HNV) grasslands (Veen et al., 2009), which are still widespread in the Transylvanian section of the Carpathian Mountains (Huband et al., 2010). Compared to Western Europe, however, there is a large gap of knowledge concerning the abundance and diversity of cavity‐nesting Hymenopterans in Eastern Europe. Up to this date, only a few studies have taken on this topic in Eastern Europe (e.g., Budrys et al., 2010) and no study has addressed this issue in Transylvania. This highlights the need for more studies from such less‐disturbed reference landscapes.

Therefore, the goals of our present pilot study were the following: (a) to assess and quantify the abundance and taxon diversity of the cavity‐nesting Hymenopteran assemblage in our study area; |(b) to identify and quantify the spider taxa preyed by the spider‐hunting representatives of the Hymenopteran taxa; (c) to analyze the influence of the proportion and edge density of low‐intensity agricultural areas around the study sites on both Hymenopteran and spider prey taxa. Concerning our first goal, we were interested if we would encounter a different taxon composition of cavity‐nesting Hymenopterans in the rural, low‐intensity agricultural landscape of our study area compared to other, more intensively used Western European study areas (Table 1). Regarding our last goal, we were curious to find out which cavity‐nesting Hymenopteran and spider prey taxa would be significantly affected by the proportion and edge density of low‐intensity agricultural areas around our study sites.

**TABLE 1:** Examples for studies with a similar study design, analyzing the abundance and diversity of cavity‐nesting Hymenopterans, carried out in different Western European countries

Reference	Country	Landscape	Sites	Trap nests	Reeds	Sampling period	Most abundant taxa
Albrecht et al. (2007)	Switzerland	Grassland	13	8	ca. 200	April–October	*Trypoxylon figulus*
Steffan‐Dewenter (2002)	Germany	Agricultural	15	8	150–180	April–October	*Osmia bicornis (rufa)*; *Hylaeus communis*
Diekötter et al. (2014)	Germany	Agricultural	12	2	NA	March–October	*Osmia bicornis (rufa)*
Fabian et al. (2013) and Fabian et al. (2014)	Switzerland	Agricultural	12	14	170–180	April–October	*Osmia bicornis*; *Trypoxylon figulus*; *Ancistrocerus nigricornis*
Gathmann et al. (1994)	Germany	Agricultural	40	6	180	April–October	*Megachile* sp.; *Osmia* sp.; *Trypoxylon* sp.
Happe et al. (2018)	Germany	Agricultural	36	2	NA	April–September	
Holzschuh et al. (2009)	Germany	Agricultural	12	5	ca. 200	April–September	*Trypoxylon* sp.; *Symmorphus* sp.
Holzschuh et al. (2010)	Germany	Agricultural	46	2	150–180	April–July	*Osmia bicornis (rufa)*
Krewenka et al. (2011)	Germany	Grassland	55	216 (total)	ca. 200	April–October	*Trypoxylon* sp.; *Passaloecus* sp.
Kruess and Tscharntke (2002)	Germany	Grassland	18	4	150–180	April–October	*Trypoxylon figulus*
Schüepp et al. (2011)	Switzerland	Agricultural	30	2	ca. 170	April–October	*Trypoxylon figulus*; *Osmia bicornis*
Sobek et al. (2009)	Germany	Woodland	12	12	NA	May–September	*Ancistrocerus trifasciatus*; *Trypoxylon clavicerum*

Note

We distinguished three different groups of studies according to the main characteristic of the landscape around the study sites in these studies (=agricultural landscape, grassland, woodland). The number of sites, trap nests per site, reeds (*Phragmites australis* Cav.), the sampling period, and the most abundant taxa reported in these studies are also given. The reed diameters, if reported, ranged from 2 to 10 mm in almost every case.

## 2 MATERIALS AND METHODS

### 2.1 Study sites

**FIGURE 1:**
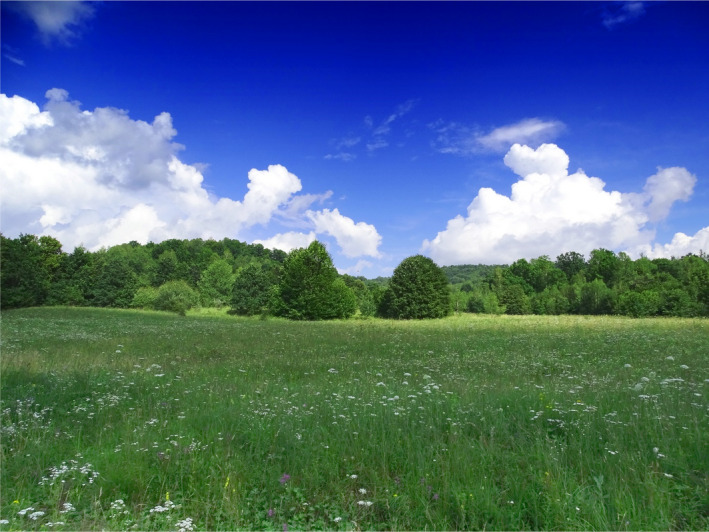
Typical landscape in the study area

The study took place in a hilly mountainous area at the border of the two counties Hargita and Kovászna (Transylvania, Romania), where the valleys are predominantly used for extensive, small‐scale farming. The landscape surrounding our study sites can be defined as a cultural‐historic low‐intensity agricultural landscape, which consists of a mosaic of grassland and woodland patches. The grassland patches are mostly used as meadows and pastures, where grazing is made with low numbers of cattle and predominantly hand‐mowing is applied (Figure 1). The eight study sites were located in three valleys between 530 and 630 m a.s.l. (Figure S1). The natural vegetation in this region at this sea level mostly consists of sessile oak‐hornbeam or hornbeam‐sessile oak (Querco petraeae‐Carpinetum or Carpino‐Quercetum petraeae) and hornbeam‐beech or bastard balm‐beech (Carpino‐Fagetum or Melittio‐Fagetum) mixed forests (Benke, 2004; Szabó, 1985). Two of these valleys were formed by the Vargyas creek (=‘Vargyas valleys’) and are separated by a canyon (Figure S1A). The third one is located 5–8 km east to the Vargyas valleys and was formed by the Körmös creek (=‘Körmös valley’; Figure S1B). The main flow direction of both creeks in this area is north to south. The Northern Vargyas valley is mostly used for extensive grazing and is dominated by meadows and pastures, while the Southern Vargyas valley, due to its remoteness, is much less used for grazing and more dominated by forest patches. Compared to the two Vargyas valleys, the Körmös valley is more strongly influenced by humans with arable land in its southern part, close to the settlement Erdőfüle (Filia). As a result of these differences in the intensity of land use, the ratio of low‐intensity agricultural areas to the natural woodland and other areas in the close surroundings of the eight study sites also differed from site to site (Table S1). We established three sites each in the Körmös valley (K1–K3) and Southern Vargyas valley (SV1–SV3) and two sites in the Northern Vargyas valley (NV1–NV2). The selection of the sites happened randomly, only paying attention to that the center points of each site should be at least 500 m away from each other. As the majority of the study sites were located within the borders of three Natura 2000 sites (ROSPA0027, ROSCI0036, and ROSCI0091), the number of sampling sites as well as the intensity of the sampling procedure was limited. Natura 2000 sites belong to a large, coordinated network of protected areas in the European Union, which were selected and established with the aim to ensure the long‐term survival of threatened species and valuable habitats, listed under both the Birds Directive and the Habitats Directive of the European Commission (European Commission, 2021).

### 2.2 Trap nests

**FIGURE 2:**
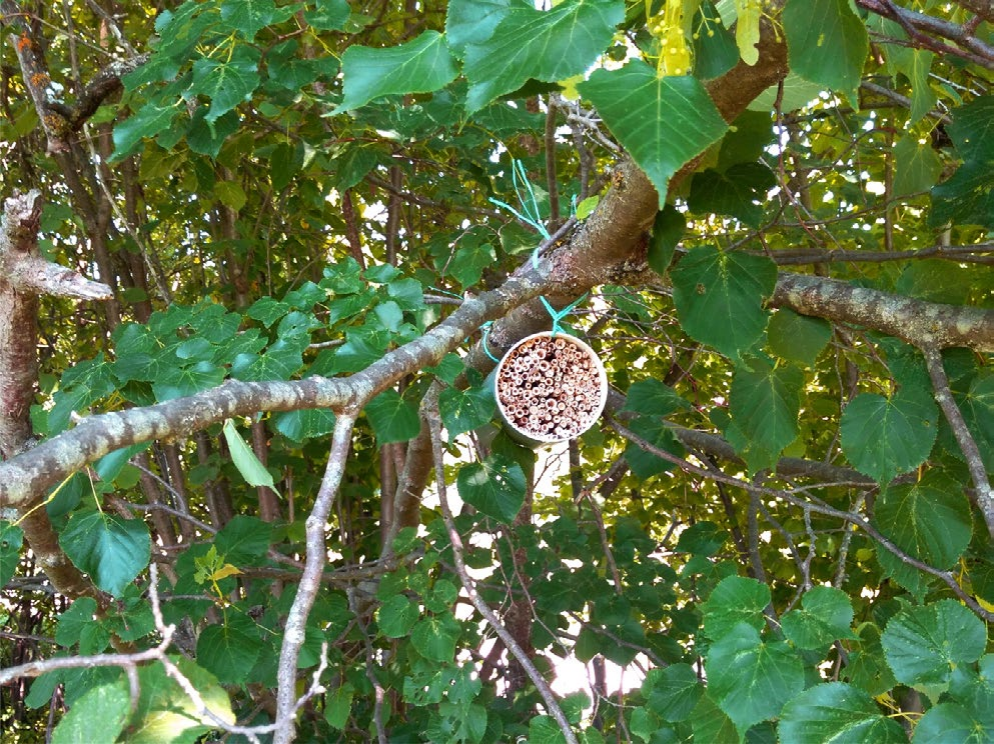
A trap nest, mounted to a tree branch

We installed four trap nests each at the eight study sites at the end of May 2018 (Figure S1). All trap nests were marked with a unique code in reference to the sites and placed within 100 m distance around the center point. The trap nests were custom‐made, consisting of a PVC tube of 12 cm diameter and 23 cm length (Figure 2). The tubes were filled with stalks of common reed (*Phragmites australis* Cav.), which were cut off to a length of approx. 22 cm between the nodes, so that the inner part of the stalks would be freely accessible for any nest‐building Hymenopteran. The stalks were placed tightly packed in the tubes to avoid them from falling out. The tubes were placed in trees or shrubs at 1–2 m above ground. The trap nests were collected at the end of August 2018 and stored outdoors at a shady place. In January 2019, the nests were put into a refrigerator and stored at 4–7°C. In the same month, we began to collect the data from the reed stalks. For this, all stalks were cut open, and, in case we found a nest within a stalk, it was recorded with reference to the unique code of the trap nest plus a serial number, giving each nest a unique ID code. In case of each occupied stalk (=nest), we recorded the following parameters: (a) diameters of the reed stalks; (b) number of occupied brood cells, filled either with Hymenopteran offspring or spider prey (if present)—empty cells were also counted, but not used in further analyses; (c) type of nesting material; (d) color of larvae or cocoons (if present). Besides these parameters, we also counted the total number of stalks per trap nest. Based on the parameters (c) and (d), we were able to identify seven groups of nest types. From each of these seven groups, we also took a few nest samples (at least two) and reared them at room temperature in plastic bags. After the emergence of the adults from these samples, at least two specimens from each nest sample were collected, killed in 70% ethanol, and identified at genus level. We were able to identify the following eight genera: *Ancistrocerus*, *Auplopus*, *Dipogon*, *Hylaeu*s, *Megachile*, *Osmia*, *Symmorphus,* and *Trypoxylon*. Except for the two genera *Ancistrocerus* and *Symmorphus* of the subfamily of Eumeninae (potter wasps), which could not be distinguished based on the nest type, each genus was assigned to a specific nest type. Therefore, based on this information, we distinguished between three taxa of solitary bees and four taxa of predatory, solitary wasps, giving them the name of the respective genus, except for the two genera of potter wasps, which were named after the subfamily.

If present, spider prey specimens were collected from the nests, put into 70% ethanol, and marked with the unique nest ID codes. The spider prey were then taxonomically identified at species level—if possible, but at least at family level—and grouped according to the taxon of the spider‐hunting wasp and the identified spider families.

### 2.3 Landscape context

The landscape surrounding the eight study sites was mapped as landscape sectors of 250 m radius in QGIS 2.18.9 (QGIS Development Team 2009) in the ETRS89/ETRS‐LAEA (EPSG: 3035) coordinate reference system. We distinguished between three different landscape element types: (a) ‘low‐intensity agricultural areas’ like meadows, pastures, and small patches of arable land (small‐scale farming); (b) ‘woodland’; and (c) ‘other areas’, like the water bodies of the two creeks, the creek banks without vegetation, as well as dirt roads. The categories of ‘woodland’ and ‘other areas’ were not included in further analyses. We decided to calculate the landscape metrics ‘Percentage of Landscape’ and ‘Edge Density’ in FRAGSTATS v4.2.1 (McGarigal et al., 2002) to quantify the landscape structure around the eight study sites (Table S1). We chose these two metrics due to their common use in landscape analysis and their easy interpretability. For calculating the landscape metrics, the vector layers of the landscape sectors were rasterized with an output raster cell size of 1 × 1 m. We used an 8‐cell neighbor‐hood rule for all calculations carried out with FRAGSTATS v4.2.1. The calculated values for the proportion and edge density of the low‐intensity agricultural areas within 250 m around the eight study sites are listed in the Table S1.

### 2.4 Statistical analyses

All statistical analyses were conducted in R v3.6.3 (R Core Team, 2020), and all graphs were created using the R package ‘ggplot2’ (Wickham, 2016). The relationship between the nest numbers of solitary wasp and bee taxa was tested with a generalized linear model (GLM) assuming a Poisson distribution. We conducted principal component analyses (PCAs) using functions from the R packages ‘FactoMineR’ (Le et al., 2008) and ‘factoextra’ (Kassambara & Mundt, 2020). These PCAs were used to reveal if there was a relationship between the study sites and the nest numbers of the Hymenopteran taxa and the specimen numbers of the most commonly preyed spider families, that is the families of Araneidae, Linyphiidae, and Theridiidae for *Trypxylon* (all above 100 specimens) as well as Thomisidae, which was the most frequently preyed spider family for *Dipogon*. All variables were scaled prior to the PCAs. The differences in the reed stalks' diameter used by the Hymenopteran taxa for nesting were tested with an ANOVA followed by a post hoc Tukey's HSD test (confidence level = 0.95). The relationship between the number of nests and occupied brood cells for the seven cavity‐nesting Hymenopteran taxa was tested with linear models (LMs).

We applied generalized linear mixed models (GLMMs) assuming a Poisson distribution from the R package ‘lme4’ (Bates et al., 2015) to analyze the effects of the proportion and edge density of the low‐intensity agricultural areas on the cavity‐nesting Hymenopteran taxa and the most commonly preyed spider families. In these GLMMs, we used the number of occupied brood cells (=parameter b) per site for all seven cavity‐nesting Hymenopteran taxa and the number of preyed spider specimens per site for the most frequently preyed spider families. The number of occupied brood cells was chosen, because it showed a considerably higher variance than the number of nests for rarer taxa. The IDs of the eight study sites were included as a random effect in all GLMMs. The metrics of low‐intensity agricultural areas were scaled prior to the GLMMs.

The relationship between the proportion and edge density of the low‐intensity agricultural areas and the diversities per site for both Hymenopteran taxa and *Trypoxylon* spider prey was analyzed with linear models (LMs). The diversity of both groups was assessed by calculating Shannon's Diversity Indices (SDIs) using the R package ‘vegan’ version 2.5‐6. (Oksanen et al., 2019). The SDIs were determined using the number of occupied brood cells per site for the Hymenopteran taxa and the number of spider specimens per site for the spider families preyed by *Trypoxylon*. For the *Trypoxylon* spider prey diversity, the specimen numbers from all seven identified spider families were used for determining the SDI. The distribution of both SDIs fulfilled the assumption of normality. The metrics of low‐intensity agricultural areas were scaled prior to the LMs.

The residuals of all LMs, GLMs, and GLMMs were tested for uniformity, dispersion, and outliers using functions from the R package ‘DHARMa’ (Hartig, 2020). We did not detect any significant deviations for the residuals of the tested models. Finally, we also checked for spatial autocorrelation (Moran's I) in the case of those data, where we encountered a significant effect of the landscape context, using the R package ‘ape’ (Paradis & Schliep, 2019). The coordinate reference system used for this analysis was ETRS89/ETRS‐LAEA (EPSG: 3,035), the same one as used for mapping. We only detected significant spatial autocorrelation in the case of the brood cells of the genus *Megachile* (Table S2). Therefore, besides the normal linear regression models, we also used generalized least squares fits (‘gls’) by REML from the R package ‘nlme’ (Pinheiro et al., 2013), incorporating a Gaussian correlation structure in order to account for the spatial autocorrelation in case of *Megachile*. The brood cell numbers of *Megachile* were “log+1”‐transformed for this analysis.

## 3 RESULTS

### 3.1 Nests

In total, we found 990 nests in 4,857 reed stalks, with the occupancy per site ranging from ca. 13%–30% with a mean number of 20 ± 6% for all sites (Table S3A). The majority of the nests were built by solitary wasps (*n* = 888; Table S3B), with the genus *Trypoxylon* (*n* = 560) being the most abundant nest‐building taxon at five of the eight study sites, especially at those located in the Southern Vargyas valley (SV1–SV3). We found 158 nests built by the wasp genus *Dipogon*, which was the most abundant nest‐building taxon at two study sites (K3 and NV1). The nests of *Dipogon* occurred at all sites, but always in a balanced manner with nest numbers ranging between 8 and 27 (19.75 ± 6.76 nests per site on average). We identified 152 nests built by representatives of the subfamily of potter wasps (Eumeninae). The nests of potter wasps were found at all sites, but with strongly varying numbers, ranging from the most abundant nest‐building taxon at one site (K1 with 51 nests) to nearly absent at another site (K3 with 2 nests). Nests of the wasp genus *Auplopus* occurred at seven of the eight study sites, but always with very low numbers (*n* = 18 in total).

**FIGURE 3:**
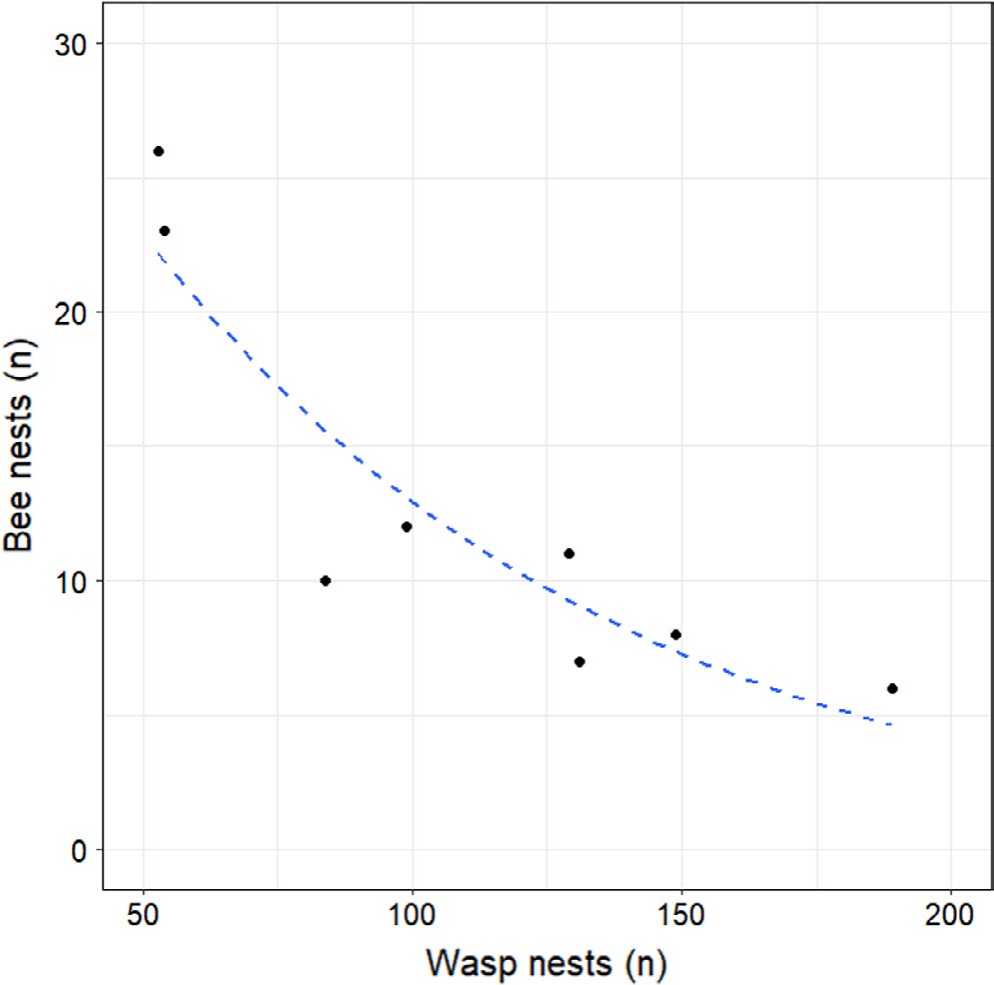
Relationship between the number of wasp and bee nests at the eight study sites. The dashed blue line represents a generalized linear model (GLM) assuming a Poisson distribution, fitted to the data points

The number of nests built by solitary bees was relatively low compared to those built by solitary wasps (*n* = 102; Table S3B). We found a total of 61 nests built by the genus *Hylaeus*, followed by the genera *Osmia* (*n* = 23) and *Megachile* (*n* = 18). From these solitary bee genera, only nests built by *Hylaeus* were occurring at all sites. We also observed that an increasing number of wasp nests encountered at a study site had a significantly negative effect (Estimate = −0.01; *df* = 6; *z* value = −4.61; *p*‐value < 0.001) on the number of bee nests (Figure 3).

**FIGURE 4:**
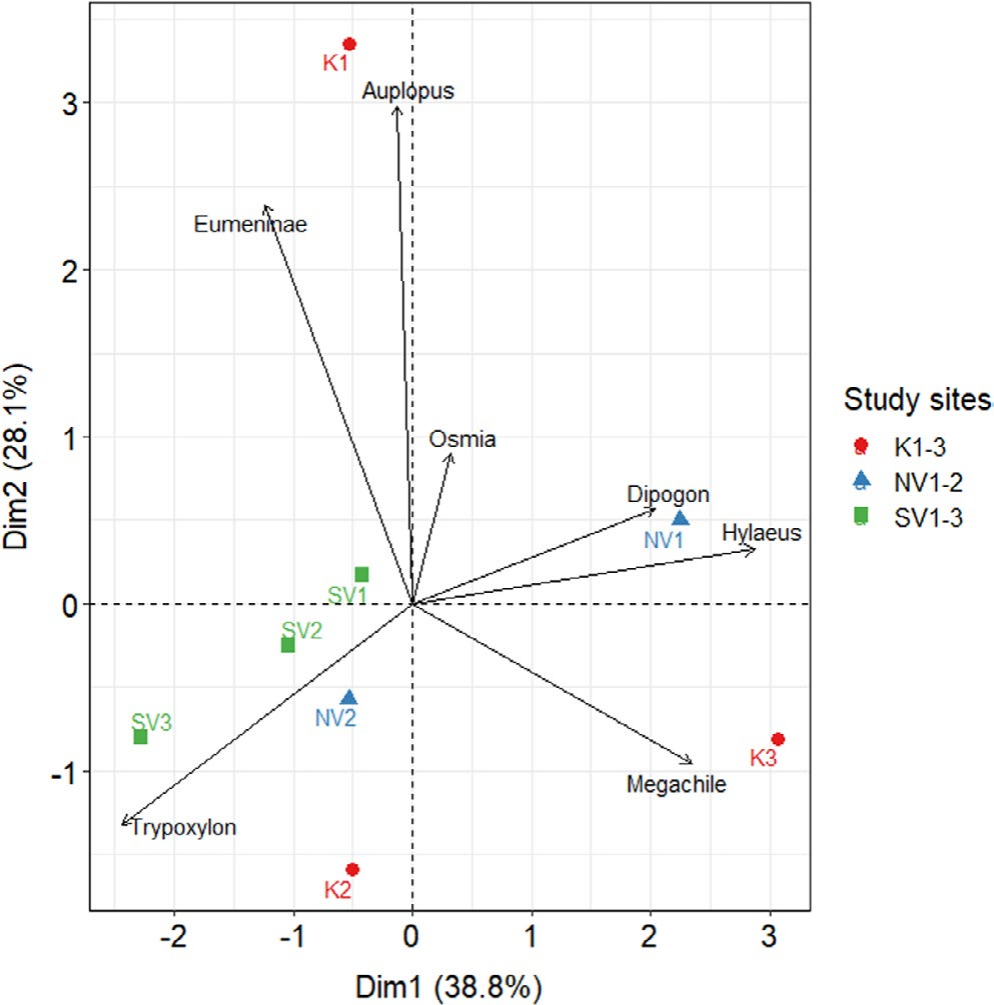
Principal component analysis (PCA) biplot of the nest numbers of the cavity‐nesting Hymenopteran taxa per site. The length of the arrows represents the strength of the association with the study sites

The PCA (Figure 4) conducted on the nest data also indicated that the nest numbers of *Trypoxylon* were most strongly associated with the study site SV3 and less strongly with four other study sites (K2, NV2, SV1, and SV2). The nest numbers of the other cavity‐nesting Hymenopteran taxa were most strongly associated each with one site (*Dipogon*, *Hylaeus* with NV1; *Auplopus* and Eumeninae with K1; *Megachile* with K3). Only the nests of *Osmia* had no clear association with any site.

We partially found differences in the diameters of the reed stalks, which the Hymenopteran taxa used for nesting (Figure  6). Overall, the occupied reed stalks (=nests) had a mean diameter of 6.62 ± 0.33 mm (Table S3A). The genus of the small‐sized solitary bee *Hylaeus* built its nests in stalks with the smallest diameters (5.68 ± 0.75 mm). The three most common wasp taxa found in the trap nests—*Trypoxylon*, *Dipogon,* and Eumeninae—all choose reed stalks of very similar diameters, which were close to the overall mean diameter of all reeds with nests inside (6.57 ± 1.02 mm for *Trypoxylon*; 6.45 ± 0.82 mm for *Dipogon*; 6.62 ± 1.19 mm for Eumeninae). The two medium‐sized solitary bee genera *Osmia* and *Megachile*, as well as the Pompilid wasp *Auplopus*, which builds nests with barrel‐shaped cells, all favored reed stalks with larger diameters: *Osmia* (7.45 ± 1.40 mm), *Megachile* (8.44 ± 1.19 mm), and *Auplopus* (8.21 ± 0.89 mm).

Regarding the number of occupied brood cells per site (Table S3C), we found significant relationships between the number of nests and cells for all Hymenopteran taxa (Table S4A). The genus *Dipogon* had the lowest mean number of cells per nest (<3), while *Auplopus* and *Osmia* had the highest numbers of cells per nest (>4) from all Hymenopteran taxa (Table S4B).

### 3.2 Spider prey

The largest number of identifiable spiders was preyed by *Trypoxylon* with a total number of 1,471 specimens (Table S5A), followed by *Dipogon* with 99 identifiable specimens (Table S5B) and *Auplopus* with only one identifiable specimen from the family of Clubionidae at the NV1 site. The majority of spiders preyed by *Trypoxylon* were from the family of Araneidae (*n* = 1,118). Among Araneidae, *Mangora acalypha* was the most abundant species, occurring in 14 different nests (*n* = 17). Other spider families, which were more commonly preyed by *Trypoxylon*, were the Linyphiidae (*n* = 175), with *Linyphia triangularis* as the most abundant species encountered in 18 different nests (*n* = 44), and the Theridiidae (*n* = 131), with *Phylloneta impressa* as the most common species found in 14 different nests (*n* = 53). Other spider families preyed by *Trypoxylon* were the Tetragnathidae (*n* = 31), Thomisidae (*n* = 10), Salticidae (*n* = 4), and Trachelidae (*n* = 1). *Dipogon* clearly differed in its prey use from *Trypoxylon*, with mostly preying on spiders from the family of Thomisidae (*n* = 93). The most abundant species from this family found in *Dipogon* nests were *Xysticus bifasciatus* (*n* = 4) and *Xysticus cristatus* (*n* = 4). The PCA (Figure 6) conducted on the numbers of the four most common spider prey families of *Trypoxylon* and *Dipogon* showed that the Araneid prey of *Trypoxylon* and Thomisid prey of *Dipogon* were mostly related to the study sites in the Southern Vargyas valley (SV1‐SV3), whereas the Linyphiid and Theridiid prey of *Trypoxylon* were strongly associated with the K2 site.

### 3.3 Low‐intensity agricultural areas

The cell numbers of the Hymenopteran taxa of *Auplopus*, *Megachile,* and *Osmia* were significantly correlated with both the edge density and proportion of low‐intensity agricultural areas around the study sites (Table 2). The strongest, significant relationships with low‐intensity agricultural areas were found for *Osmia*, where an increasing edge density and proportion of these areas both had negative effects on the cell numbers of this bee genus (Table 2). The cell numbers of the *Auplopus* wasp genus were significantly, positively correlated with an increasing edge density and negatively with an increasing proportion of low‐intensity agricultural areas (Table 2). The cell numbers of the *Megachile* bee genus were significantly, positively correlated with both an increasing edge density as well as an increasing proportion of low‐intensity agricultural areas (Table 2). However, the effects of both the edge density (Estimate = 0.01; *t*‐value = 1.80; *p*‐value = 0.12) and proportion of low‐intensity agricultural areas (Estimate = 0.02; *t*‐value = 0.60; *p*‐value = 0.57) were not significant in the models corrected for spatial autocorrelation.

**TABLE 2:** Results of generalized linear mixed models (GLMMs) assuming a Poisson distribution, testing for the relationship between the proportion and edge density of low‐intensity agricultural areas within 250 m around the eight study sites and the total number of occupied brood cells per nest and site, built by different cavity‐nesting Hymenopteran taxa

Metric	Taxon	Estimate	*SE*	*z* value	Pr(>|*z*|)
Edge density	Bees	0.00	0.00	0.00	1.00
	Wasps	0.00	0.00	−1.33	0.18
	*Auplopus*	0.01	0.00	**1.99**	**0.05**
	*Dipogon*	0.00	0.00	−0.29	0.77
	Eumeninae	0.00	0.01	−0.15	0.88
	*Hylaeus*	0.01	0.01	1.31	0.19
	*Megachile*	0.02	0.01	**2.06**	**0.04**
	*Osmia*	−0.01	0.00	**−2.15**	**0.03**
	*Trypoxylon*	−0.01	0.01	−1.57	0.12
Proportion	Bees	0.00	0.01	0.03	0.97
	Wasps	−0.02	0.01	−1.89	0.06
	*Auplopus*	−0.02	0.01	**−3.05**	**0.00**
	*Dipogon*	−0.02	0.01	−1.28	0.20
	Eumeninae	−0.03	0.03	−0.98	0.33
	*Hylaeus*	0.02	0.02	0.64	0.52
	*Megachile*	0.08	0.04	**2.18**	**0.03**
	*Osmia*	−0.03	0.00	**−6.61**	**0.00**
	*Trypoxylon*	−0.03	0.02	−1.30	0.20

Note

The IDs of the sites were included as a random effect in these GLMMs. The number of observations was 8 in each case. Significant relationships are marked bold.

The number of *Trypoxylon* and *Dipogon* spider prey was largely unaffected by the edge density and proportion of low‐intensity agricultural areas around the study sites (Table 3). From the preyed spider families, only the numbers of Thomisidae were significantly, negatively correlated with an increasing proportion of low‐intensity agricultural areas (Table 3).

**TABLE 3:** Results of generalized linear mixed models (GLMMs) assuming a Poisson distribution, testing for the relationship between the proportion and edge density of low‐intensity agricultural areas within 250 m around the eight study sites and the total number of spider specimens per nest and site from families, which were most commonly preyed by the wasp genera *Trypoxylon* (Try) and *Dipogon* (Dip)

Metric	Family	Estimate	*SE*	*z* value	Pr(>|*z*|)
Edge density	Araneidae (Try)	−0.02	0.01	−1.84	0.07
	Linyphiidae (Try)	0.00	0.01	−0.23	0.82
	Theridiidae (Try)	−0.01	0.01	−1.08	0.28
	Thomisidae (Dip)	0.00	0.01	−0.57	0.57
Proportion	Araneidae (Try)	−0.07	0.04	−1.70	0.09
	Linyphiidae (Try)	0.01	0.04	0.40	0.69
	Theridiidae (Try)	0.01	0.05	0.19	0.85
	Thomisidae (Dip)	−0.06	0.01	**−9.41**	**0.00**

Note

The IDs of the sites were included as a random effect in these GLMMs. The number of observations was 8 in each case. Significant relationships are marked bold.

The edge density and proportion of low‐intensity agricultural areas had no significant effects on the SDI of the nest‐building solitary Hymenopteran taxa at the study sites (Table 4). The SDI of the *Trypoxylon* spider prey, however, was significantly, positively influenced by the proportion of low‐intensity agricultural areas around the study sites (Table 4).

**TABLE 4:** Results of linear models (LMs) testing for the relationship between the proportion and edge density of low‐intensity agricultural areas within 250 m around the eight study sites and the Shannon's Diversity Indices (SDIs) per site of the Hymenopteran taxa and *Trypoxylon* spider prey

Diversity	Metric	Estimate	*SE*	*t*‐value	Pr(>|*t*|)
Hymenopteran	Edge density	0.00	0.00	1.17	0.29
	Proportion	0.00	0.00	1.37	0.22
Spider prey	Edge density	0.01	0.01	0.62	0.56
	Proportion	0.03	0.01	**4.38**	**0.00**

Note

For the calculation of the SDIs for the *Trypoxylon* spider prey, representatives of all spider families preyed by *Trypoxylon* (Table S5A) were included. The number of observations was 8 in each case. Significant relationships are marked bold.

## 4 DISCUSSION

### 4.1 Nests

**FIGURE 5:**
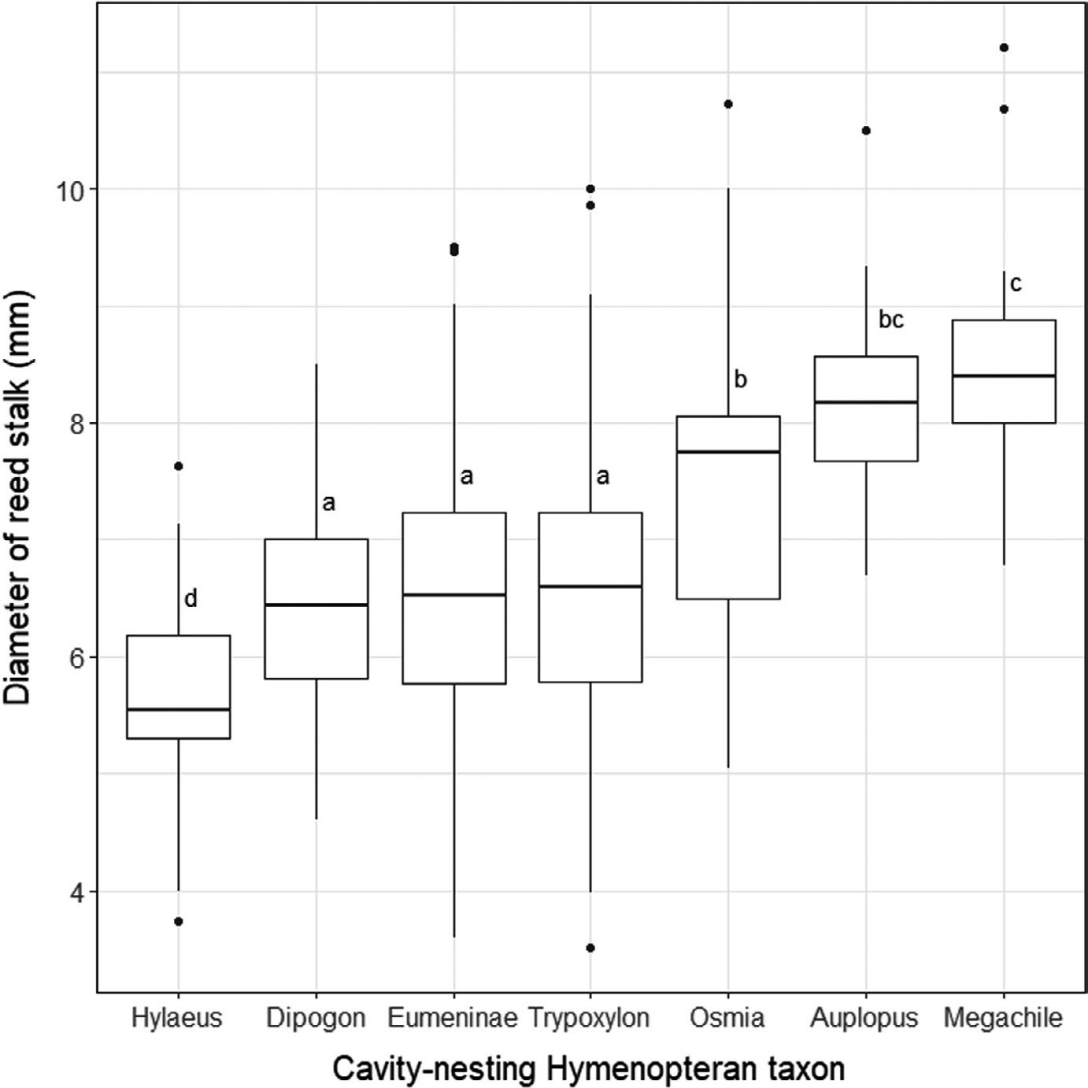
Diameter of the reed stalks with nests for the seven cavity‐nesting Hymenopteran taxa found at our study sites. The horizontal lines indicate the median value. The lower and upper whiskers represent the maximum values of the data that are within 1.5 times the interquartile range under the 25th and over the 75th percentile, respectively. Outlier values, indicated by black dots, are any values under or over this range. Same letters indicate no statistical differences between groups (Tukey's HSD test, *p* < 0.05)

Analyzing the content of the trap nests revealed that the nest numbers of solitary wasps were higher than the nest numbers of solitary bees across all study sites. Nests built by solitary bees were only more frequently found at two sites (K3 and NV1), where the numbers of wasp nests were relatively low. From these two sites, NV1 was located the closest to the border of the Natura 2000 site ‘ROSPA0027’ framing the two Vargyas valleys and K3 was completely situated outside the Natura 2000 site ‘ROSCI0091’, which extends over the eastern part of the Körmös valley (Figure S1A and B). Thus, an increasing nest number of solitary bees might be the indication of an increasing human impact at the study sites. Solitary bees were also the most abundant taxa in the majority of those Western European studies that were conducted in high‐intensity agricultural landscapes (Table 1), whereas in studies, that were carried out in natural (Sobek et al., 2009) or low‐intensity agricultural (Albrecht et al., 2007; Krewenka et al., 2011; Kruess & Tscharntke, 2002) landscapes with higher proportions of grass‐ or woodland, solitary wasp taxa were the most abundant nesting taxa. The results of the PCAs also indicated that the occurrence of the most abundant cavity‐nesting Hymenopteran taxon *Trypoxylon* showed the strongest association with study sites located in the Southern Vargyas valley, where human disturbance is relatively low. Another possible explanation for low numbers of bee nests is that competitive pressure from higher wasp densities caused solitary bee taxa to search for alternative nesting locations. This theory is partially supported by the reed diameters chosen for the nests of the most common solitary wasp taxa (=*Trypoxylon*, *Dipogon,* and Eumeninae; Figure 5), which were very close to the overall mean diameter of all reeds with nests inside (=6.62 ± 0.33 mm). This indicates that reed stalks with average diameters were preferably occupied by the most abundant Hymenopteran taxa, leaving the other, rarer taxa only stalks with much smaller or larger diameters for nesting (Figure 5). Using a specific reed diameter for nesting, however, could also be related to the body size or proportions of the Hymenopteran taxa. For example, the smallest taxon *Hylaeus* also choose reeds with the smallest diameters for its nests (Figure 5). It is also possible that the sampling period of our study did not overlap well with the breeding time of the local solitary bee taxa. The results of another study using sweep‐net methods, conducted parallel to this one during 2018 in the same area, support this theory as they revealed that the occurrence of *Osmia* species was mainly in spring (April and May), while their occurrence between June and August, the time when the trap nests were available for them, was considerably lower (Demeter et al., 2021).

### 4.2 Spider prey

**FIGURE 6:**
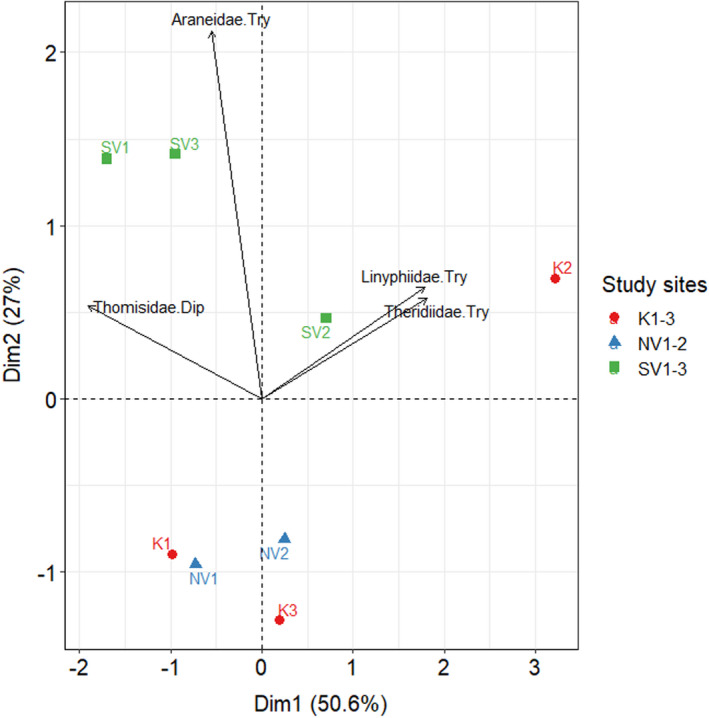
Principal component analysis (PCA) biplot of the numbers of *Trypoxylon* (Try) and *Dipogon* (Dip) spider prey per site. The length of the arrows represents the strength of the association with the study sites

We found that the majority of spider specimens preyed by the genus *Trypoxylon* were from the family of Araneidae. In contrast to our findings, however, two other studies reported that the majority of spider specimens preyed by *Trypoxylon figulus* were from the family of Theridiidae (Coudrain et al., 2013; Hoffmann et al., 2020). A possible explanation for the different findings of these two studies is that they were carried out in more intensively used agricultural landscapes. The results of the PCAs also support this assumption as they indicate that the Araneid prey of *Trypoxylon* was closely related to the study sites located in the remote Southern Vargyas valley (SV1‐SV3), where the proportions of low‐intensity agricultural areas were considerably lower than at the other study sites. However, the Theridiid prey of *Trypoxylon* was strongly associated with the study site K2, where low‐intensity agricultural areas were the proportionally most dominant landscape element (Figure 6).

### 4.3 Low‐intensity agricultural areas

The brood cell numbers of *Osmia* were significantly lower at study sites with both a higher edge density and proportion of low‐intensity agricultural areas. This finding may come a bit unexpected, since most *Osmia* species feed on wild flowers, but many species are closely associated with forest habitats due to their nesting habits as they create small burrows for their nests in tree barks (Müller et al., 2019). In contrast to *Osmia*, a higher edge density and proportion of low‐intensity agricultural areas both had a significantly positive effect on the brood cell numbers of *Megachile*. The brood cell numbers of the Pompilid were *Auplopus* were positively correlated with an increasing edge density, but negatively with an increasing proportion of low‐intensity agricultural areas. This latter finding corresponds well with those reported by Holzschuh et al. (2009), who found that the abundance of Eumenid, Pompilid, and Sphecid wasps were highest at forest edges, which provide natural nesting sites, and lowest in grass strips, with a few natural nesting sites. They also reported that wasp abundance in grass strips connected to forest edges was higher than in slightly isolated grass strips and much higher than in highly isolated grass strips.

We did not detect any significant relationship between the edge density or proportion of low‐intensity agricultural areas and the diversity of the nest‐building solitary Hymenopteran taxa. Other studies, however, found that landscape context had significant effects on Hymenopteran species diversity: Steffan‐Dewenter (2002) reported a positive relationship between an increasing proportion of semi‐natural habitats and the number of Hymenopteran species, while Schüepp et al. (2011) found that species richness of wasps was more than doubled and diversity three‐times higher at sites with high percentages of woody habitats, compared to sites with low percentages of woody habitats, and Fabian et al. (2013) also reported that forest cover had a positive effect on the species richness of wasps. All three studies were conducted in intensively managed agricultural landscapes with relatively low proportions of semi‐natural habitats, which may explain the positive effect of an increasing proportion of semi‐natural habitats on the Hymenopteran species diversity in their studies.

Regarding the spider prey of *Dipogon* and *Trypoxylon*, only the numbers of Thomisid prey specimens found in *Dipogon* nests were significantly affected by low‐intensity agricultural areas, with an increasing proportion in the studied landscape sectors having a negative effect on the number of preyed specimens. An increasing proportion of low‐intensity agricultural areas also had a significant effect on the diversity of spider prey found in the nests of *Trypoxylon*, with the diversity being higher at study sites surrounded by a higher proportion of low‐intensity agricultural areas. In other words, the lower the proportion of low‐intensity agricultural areas was around the study sites, the higher was the proportion of Araneid specimens among the spiders preyed by *Trypoxylon*, which resulted in a lower diversity of *Trypoxylon* spider prey. The highest numbers of Araneid prey were encountered at the study sites SV1 and SV3, where the proportion of low‐intensity agricultural areas was the lowest with regards to all eight study sites (Table S1). Hoffmann et al. (2020), however, reported exactly the opposite, with an increasing area of grassland having a negative effect on the species diversity of spiders in *Trypoxylon* nests. Again, this contrasting finding may be explained by the different composition and structure of the intensively managed agricultural landscape in their study area, where they found that *Trypoxylon* mostly preyed in grassland patches. Therefore, they also assumed that a higher proportion of grassland may cause *Trypoxylon* specifically hunting for its preferred prey species, resulting in a lower prey diversity found in their nests.

## 5 CONCLUSION

We encountered a considerably higher abundance of nests built by solitary wasps than solitary bees at all study sites. The two study sites with the highest numbers of solitary bee nests (=K3 and NV1) were both located the furthest away from the respective centers of the Natura 2000 protected areas. These results indicate that solitary bees are more common in areas, where the impact of human activities is stronger. In contrast to this, solitary wasps seem to rather avoid these areas. Our findings correspond well with those of similar previous studies from Western Europe, where solitary bees were the most abundant nest‐building taxa in the majority of those studies, which were conducted in high‐intensity agricultural areas. However, solitary wasps were the most abundant nest‐building taxa in most studies, which were carried out in similar low‐intensity agricultural or natural areas. Of course, this phenomenon could also be related to the chosen time period for sampling. However, as most studies from Table 1 chose similar time periods for sampling (mostly from April to October), this might not be the cause for the lower abundances of solitary wasps and higher abundances of solitary bees in high‐intensity agricultural landscapes. Therefore, we suggest that future studies not only should put more effort into sampling in reference landscapes with low‐intensity agriculture but also focus more on solitary wasp taxa, when sampling such an area. As there are only a few such landscapes in Europe still remaining and as the maintenance of Hymenopteran biodiversity is crucial for the well‐functioning of many ecosystem processes, our results can serve as a reference for future research in other areas, which are either less or more strongly influenced by humans.

## CONFLICT OF INTEREST

The authors of this article have no financial or other conflict of interest to declare.

## AUTHOR CONTRIBUTIONS

**Károly Lajos:** Conceptualization (supporting); Data curation (lead); Formal analysis (lead); Investigation (supporting); Methodology (equal); Supervision (equal); Validation (lead); Visualization (lead); Writing‐original draft (lead); Writing‐review & editing (lead). **Imre Demeter:** Conceptualization (supporting); Investigation (lead); Methodology (equal); Resources (equal); Writing‐original draft (supporting); Writing‐review & editing (supporting). **Róbert Mák:** Investigation (supporting). **Adalbert Balog:** Funding acquisition (lead); Project administration (supporting); Writing‐original draft (supporting); Writing‐review & editing (supporting). **Miklós Sárospataki:** Conceptualization (lead); Funding acquisition (lead); Methodology (equal); Project administration (lead); Resources (equal); Supervision (equal); Writing‐original draft (supporting); Writing‐review & editing (supporting).

## COMPLIANCE WITH ETHICAL STANDARDS

The manuscript is new in this form, it represents our own original work and has not been published, submitted or considered for publication elsewhere. The text, illustrations, and any other materials included in the manuscript do not infringe any existing copyright or other rights of anyone.

## Supporting information

Supplementary Material

## Data Availability

All data generated or analyzed during this study were collected by the authors of this publication. The data that support the findings of this study are available in the Supplementary Information of this article. Additional data are available from the Dryad Digital Repository: https://doi.org/10.5061/dryad.cjsxksn64.

## References

[ece37956-bib-0001] Albrecht, M., Duelli, P., Schmid, B., & Müller, C. B. (2007). Interaction diversity within quantified insect food webs in restored and adjacent intensively managed meadows. Journal of Animal Ecology, 76(5), 1015–1025. 10.1111/j.1365-2656.2007.01264.x 17714280

[ece37956-bib-0002] Babai, D., & Molnár, Z. (2014). Small‐scale traditional management of highly species‐rich grasslands in the Carpathians. Agriculture Ecosystems Environment, 182, 123–130. 10.1016/j.agee.2013.08.018

[ece37956-bib-0003] Babai, D., Tóth, A., Szentirmai, I., Biró, M., Máté, A., Demeter, L., Szépligeti, M., Varga, A., Molnár, Á., Kun, R., & Molnár, Z. (2015). Do conservation and agri‐environmental regulations effectively support traditional small‐scale farming in East‐Central European cultural landscapes? Biodiversity and Conservation, 24(13), 3305–3327. 10.1007/s10531-015-0971-z

[ece37956-bib-0004] Bates, D., Mächler, M., Bolker, B., & Walker, S. (2015). Fitting linear mixed‐effects models using lme4. Journal of Statistical Software, 67(1), 1–48. 10.18637/jss.v067.i01

[ece37956-bib-0005] Benke, J. (2004). A Homoródi erdőgondnokság erdeinek természetes felújítási lehetőségei. Thesis (PhD), Nyugat‐Magyarországi Egyetem.

[ece37956-bib-0011] Budrys, E., Budrienė, A., & Nevronytė, Ž. (2010). Dependence of brood cell length on nesting cavity width in Xylicolous solitary wasps of Genera Ancistrocerus and Symmorphus (Hymenoptera: Vespidae). Acta Zoologica Lituanica, 20(1), 68–76. 10.2478/v10043-010-0010-y

[ece37956-bib-0006] Carvalheiro, L. G., Kunin, W. E., Keil, P., Aguirre‐Gutiérrez, J., Ellis, W. N., Fox, R., Groom, Q., Hennekens, S., Landuyt, W. V., Maes, D., de Meutter, F. V., Michez, D., Rasmont, P., Ode, B., Potts, S. G., Reemer, M., Roberts, S. P. M., Schaminée, J., WallisDeVries, M. F., & Biesmeijer, J. C. (2013). Species richness declines and biotic homogenisation have slowed down for NW‐European pollinators and plants. Ecology Letters, 16(7), 870–878. 10.1111/ele.12121 23692632 PMC3738924

[ece37956-bib-0007] Coudrain, V., Herzog, F., & Entling, M. H. (2013). Effects of habitat fragmentation on abundance, larval food and parasitism of a spider‐hunting wasp. PLoS One, 8(3), e59286. 10.1371/journal.pone.0059286 23516622 PMC3597609

[ece37956-bib-0008] Coudrain, V., Rittiner, S., Herzog, F., Tinner, W., & Entling, M. H. (2016). Landscape distribution of food and nesting sites affect larval diet and nest size, but not abundance of *Osmia bicornis* . Insect Science, 23(5), 746–753. 10.1111/1744-7917.12238 25973721

[ece37956-bib-0009] Demeter, I., Balog, A., Józan, Z., & Sárospataki, M. (2021). Comparison of wild bee communities of three semi‐natural meadow habitats at Harghita‐Covasna region, Transylvania, Romania. Acta Zoologica Academiae Scientiarum Hungaricae, 67(2), 161–175. 10.17109/AZH.67.2.161.2021

[ece37956-bib-0010] Diekötter, T., Peter, F., Jauker, B., Wolters, V., & Jauker, F. (2014). Mass‐flowering crops increase richness of cavity‐nesting bees and wasps in modern agro‐ecosystems. GCB Bioenergy, 6(3), 219–226. 10.1111/gcbb.12080

[ece37956-bib-0012] European Commission (2021). Natura 2000. European Commission. ec.europa.eu/environment/nature/natura2000/

[ece37956-bib-0013] Fabian, Y., Sandau, N., Bruggisser, O. T., Aebi, A., Kehrli, P., Rohr, R. P., Naisbit, R. E., & Bersier, L.‐F. (2013). The importance of landscape and spatial structure for hymenopteran‐based food webs in an agro‐ecosystem. Journal of Animal Ecology, 82(6), 1203–1214. 10.1111/1365-2656.12103 23863136

[ece37956-bib-0014] Fabian, Y., Sandau, N., Bruggisser, O. T., Aebi, A., Kehrli, P., Rohr, R. P., Naisbit, R. E., & Bersier, L.‐F. (2014). Plant diversity in a nutshell: testing for small‐scale effects on trap nesting wild bees and wasps. Ecosphere, 5(2), art18. 10.1890/ES13-00375.1

[ece37956-bib-0015] Forister, M. L., Pelton, E. M., & Black, S. H. (2019). Declines in insect abundance and diversity: We know enough to act now. Conservation Science and Practice, 1(8), e80. 10.1111/csp2.80

[ece37956-bib-0016] Gathmann, A., Greiler, H.‐J., & Tscharntke, T. (1994). Trap‐nesting bees and wasps colonizing set‐aside fields: Succession and body size, management by cutting and sowing. Oecologia, 98(1), 8–14. 10.1007/bf00326084 28312790

[ece37956-bib-0017] Habel, J. C., Samways, M. J., & Schmitt, T. (2019). Mitigating the precipitous decline of terrestrial European insects: Requirements for a new strategy. Biodiversity and Conservation, 28(6), 1343–1360. 10.1007/s10531-019-01741-8

[ece37956-bib-0018] Habel, J. C., Ulrich, W., Biburger, N., Seibold, S., & Schmitt, T. (2019). Agricultural intensification drives butterfly decline. Insect Conservation and Diversity, 12(4), 289–295. 10.1111/icad.12343

[ece37956-bib-0019] Hallmann, C. A., Sorg, M., Jongejans, E., Siepel, H., Hofland, N., Schwan, H., Stenmans, W., Müller, A., Sumser, H., Hörren, T., Goulson, D., & de Kroon, H. (2017). More than 75 percent decline over 27 years in total flying insect biomass in protected areas. PLoS One, 12(10), e0185809. 10.1371/journal.pone.0185809 29045418 PMC5646769

[ece37956-bib-0020] Hallmann, C. A., Ssymank, A., Sorg, M., De Kroon, H., & Jongejans, E. (2021). Insect biomass decline scaled to species diversity: General patterns derived from a hoverfly community. Proceedings of the National Academy of Sciences of the United States of America, 118(2), e2002554117. 10.1073/pnas.2002554117 33431568 PMC7812780

[ece37956-bib-0021] Happe, A.‐K., Riesch, F., Rösch, V., Gallé, R., Tscharntke, T., & Batáry, P. (2018). Small‐scale agricultural landscapes and organic management support wild bee communities of cereal field boundaries. Agriculture, Ecosystems & Environment, 254, 92–98. 10.1016/j.agee.2017.11.019

[ece37956-bib-0022] Harris, A. C. (1994). *Ancistrocerus gazella* (Hymenoptera: Vespoidea: Eumenidae): A potentially useful biological control agent for leafrollers *Planotortrix octo*, *P. excessana*, *Ctenopseustis obliquana*, *C. herana*, and *Epiphyas postvittana* (Lepidoptera: Tortricidae) in New Zealand. New Zealand Journal of Crop and Horticultural Science, 22(3), 235–238. 10.1080/01140671.1994.9513832

[ece37956-bib-0023] Hartig, F. (2020). DHARMa: Residual Diagnostics for Hierarchical (Multi‐Level/Mixed) Regression Models. R package version 0.3.3.0. Retrieved from https://CRAN.R‐project.org/package=DHARMa

[ece37956-bib-0024] Hoffmann, U. S., Jauker, F., Diehl, E., Mader, V., Fiedler, D., Wolters, V., & Diekötter, T. (2020). The suitability of sown wildflower strips as hunting grounds for spider‐hunting wasps of the genus Trypoxylon depends on landscape context. Journal of Insect Conservation, 24(1), 125–131. 10.1007/s10841-019-00190-6

[ece37956-bib-0025] Holzschuh, A., Steffan‐Dewenter, I., & Tscharntke, T. (2009). Grass strip corridors in agricultural landscapes enhance nest‐site colonization by solitary wasps. Ecological Applications: A Publication of the Ecological Society of America, 19(1), 123–132. 10.1890/08-0384.1 19323177

[ece37956-bib-0026] Holzschuh, A., Steffan‐Dewenter, I., & Tscharntke, T. (2010). How do landscape composition and configuration, organic farming and fallow strips affect the diversity of bees, wasps and their parasitoids? The Journal of Animal Ecology, 79(2), 491–500. 10.1111/j.1365-2656.2009.01642.x 20015213

[ece37956-bib-0027] Huband, S., McCracken, D., & Mertens, A. (2010). Long and short‐distance transhumant pastoralism in Romania: Past and present drivers of change. Pastoralism: Research, Policy and Practice, 1, 55–71.

[ece37956-bib-0028] Kassambara, A., & Mundt, F. (2020). factoextra: Extract and visualize the results of multivariate data analyses. R package version 1.0.7. https://CRAN.R‐project.org/package=factoextra

[ece37956-bib-0029] Klein, A.‐M., Steffan‐Dewenter, I., & Tscharntke, T. (2006). Rain forest promotes trophic interactions and diversity of trap‐nesting Hymenoptera in adjacent agroforestry. Journal of Animal Ecology, 75(2), 315–323. 10.1111/j.1365-2656.2006.01042.x 16637985

[ece37956-bib-0030] Knop, E. (2016). Biotic homogenization of three insect groups due to urbanization. Global Change Biology, 22(1), 228–236. 10.1111/gcb.13091 26367396

[ece37956-bib-0031] Kratschmer, S., Petrović, B., Curto, M., Meimberg, H., & Pachinger, B. (2020). Pollen availability for the Horned mason bee (*Osmia cornuta*) in regions of different land use and landscape structures. Ecological Entomology, 45(3), 525–537. 10.1111/een.12823

[ece37956-bib-0032] Krewenka, K. M., Holzschuh, A., Tscharntke, T., & Dormann, C. F. (2011). Landscape elements as potential barriers and corridors for bees, wasps and parasitoids. Biological Conservation, 144(6), 1816–1825. 10.1016/j.biocon.2011.03.014

[ece37956-bib-0033] Kruess, A., & Tscharntke, T. (2002). Grazing intensity and the diversity of grasshoppers, butterflies, and trap‐nesting bees and Wasps. Conservation Biology, 16(6), 1570–1580. 10.1046/j.1523-1739.2002.01334.x

[ece37956-bib-0034] Le, S., Josse, J., & Husson, F. (2008). FactoMineR: An R package for multivariate analysis. Journal of Statistical Software, 25(1), 1–18. 10.18637/jss.v025.i01

[ece37956-bib-0035] Mayr, A. V., Peters, M. K., Eardley, C. D., Renner, M. E., Röder, J., & Steffan‐Dewenter, I. (2020). Climate and food resources shape species richness and trophic interactions of cavity‐nesting Hymenoptera. Journal of Biogeography, 47(4), 854–865. 10.1111/jbi.13753

[ece37956-bib-0036] McGarigal, K., Cushman, S., Neel, M., & Ene, E. (2002). FRAGSTATS: Spatial pattern analysis program for categorical maps. Computer software program produced by the authors at the University of Massachusetts. www.umass.edu/landeco/research/fragstats/fragstats.html

[ece37956-bib-0037] Merckx, T., & Van Dyck, H. (2019). Urbanization‐driven homogenization is more pronounced and happens at wider spatial scales in nocturnal and mobile flying insects. Global Ecology and Biogeography, 28, 1440–1455. 10.1111/geb.12969

[ece37956-bib-0038] Müller, A., Prosi, R., Praz, C., & Richter, H. (2019). Nesting in bark – the peculiar life history of the rare boreoalpine osmiine bee Osmia (Melanosmia) nigriventris (Hymenoptera, Megachilidae). Alpine Entomology, 3, 105–119. 10.3897/alpento.3.34409

[ece37956-bib-0039] Oksanen, J., Blanchet, F. G., Friendly, M., Kindt, R., Legendre, P., McGlinn, D., Minchin, P. R., O'Hara, R. B., Simpson, G. L., Solymos, P., Stevens, M. H. H., Szoecs, E., & Wagner, H. (2019). vegan: Community ecology package (2.5‐6) [Computer software]. https://CRAN.R‐project.org/package=vegan

[ece37956-bib-0040] Ollerton, J., Erenler, H., Edwards, M., & Crockett, R. (2014). Extinctions of aculeate pollinators in Britain and the role of large‐scale agricultural changes. Science, 346(6215), 1360–1362. 10.1126/science.1257259 25504719

[ece37956-bib-0041] Paradis, E., & Schliep, K. (2019). ape 5.0: An environment for modern phylogenetics and evolutionary analyses in R. Bioinformatics, 35(3), 526–528. 10.1093/bioinformatics/bty633 30016406

[ece37956-bib-0042] Piano, E., Souffreau, C., Merckx, T., Baardsen, L. F., Backeljau, T., Bonte, D., Brans, K. I., Cours, M., Dahirel, M., Debortoli, N., Decaestecker, E., De Wolf, K., Engelen, J. M. T., Fontaneto, D., Gianuca, A. T., Govaert, L., Hanashiro, F. T. T., Higuti, J., Lens, L., … Hendrickx, F. (2020). Urbanization drives cross‐taxon declines in abundance and diversity at multiple spatial scales. Global Change Biology, 26(3), 1196–1211. 10.1111/gcb.14934 31755626

[ece37956-bib-0043] Pinheiro, J., Bates, D., DebRoy, S. S., & Sarkar, D. (2013). Nlme: Linear and nonlinear mixed effects models. R Package Version 31‐110, 3, 1–113.

[ece37956-bib-0044] R Core Team (2020). R: A language and environment for statistical computing. R Foundation for Statistical Computing. https://www.Rproject.org/

[ece37956-bib-0045] Raven, P., & Wagner, D. (2021). Agricultural intensification and climate change are rapidly decreasing insect biodiversity. Proceedings of the National Academy of Sciences of the United States of America, 118, e2002548117. 10.1073/pnas.2002548117 33431564 PMC7812793

[ece37956-bib-0046] Sánchez‐Bayo, F., & Wyckhuys, K. A. G. (2019). Worldwide decline of the entomofauna: A review of its drivers. Biological Conservation, 232, 8–27. 10.1016/j.biocon.2019.01.020

[ece37956-bib-0047] Sánchez‐Bayo, F., & Wyckhuys, K. A. G. (2021). Further evidence for a global decline of the entomofauna. Austral Entomology, 60(1), 9–26. 10.1111/aen.12509

[ece37956-bib-0048] Scherber, C., Eisenhauer, N., Weisser, W. W., Schmid, B., Voigt, W., Fischer, M., Schulze, E.‐D., Roscher, C., Weigelt, A., Allan, E., Beßler, H., Bonkowski, M., Buchmann, N., Buscot, F., Clement, L. W., Ebeling, A., Engels, C., Halle, S., Kertscher, I., … Tscharntke, T. (2010). Bottom‐up effects of plant diversity on multitrophic interactions in a biodiversity experiment. Nature, 468(7323), 553–556. 10.1038/nature09492 20981010

[ece37956-bib-0049] Schüepp, C., Herrmann, J. D., Herzog, F., & Schmidt‐Entling, M. (2011). Differential effects of habitat isolation and landscape composition on wasps, bees, and their enemies. Oecologia, 165(3), 713–721. 10.1007/s00442-010-1746-6 20730546

[ece37956-bib-0050] Sobek, S., Tscharntke, T., Scherber, C., Schiele, S., & Steffan‐Dewenter, I. (2009). Canopy vs. understory: Does tree diversity affect bee and wasp communities and their natural enemies across forest strata? Forest Ecology and Management, 258(5), 609–615. 10.1016/j.foreco.2009.04.026

[ece37956-bib-0051] Staab, M., Pufal, G., Tscharntke, T., & Klein, A.‐M. (2018). Trap nests for bees and wasps to analyse trophic interactions in changing environments—A systematic overview and user guide. Methods in Ecology and Evolution, 9(11), 2226–2239. 10.1111/2041-210X.13070

[ece37956-bib-0052] Stangler, E. S., Hanson, P. E., & Steffan‐Dewenter, I. (2015). Interactive effects of habitat fragmentation and microclimate on trap‐nesting Hymenoptera and their trophic interactions in small secondary rainforest remnants. Biodiversity and Conservation, 24(3), 563–577. 10.1007/s10531-014-0836-x

[ece37956-bib-0053] Steckel, J., Westphal, C., Peters, M. K., Bellach, M., Rothenwoehrer, C., Erasmi, S., Scherber, C., Tscharntke, T., & Steffan‐Dewenter, I. (2014). Landscape composition and configuration differently affect trap‐nesting bees, wasps and their antagonists. Biological Conservation, 172, 56–64. 10.1016/j.biocon.2014.02.015

[ece37956-bib-0054] Steffan‐Dewenter, I. (2002). Landscape context affects trap‐nesting bees, wasps, and their natural enemies. Ecological Entomology, 27(5), 631–637. 10.1046/j.1365-2311.2002.00437.x

[ece37956-bib-0055] Szabó, A. (1985). A növénytakaró. In J. Péntek, & A. Szabó (Eds.), Ember és növényvilág. Kalotaszeg növényzete és népi növényismerete. Kriterion Könyvkiadó.

[ece37956-bib-0056] Taki, H., Viana, B. F., Kevan, P. G., Silva, F. O., & Buck, M. (2008). Does forest loss affect the communities of trap‐nesting wasps (Hymenoptera: Aculeata) in forests? Landscape vs. local habitat conditions. Journal of Insect Conservation, 12(1), 15–21. 10.1007/s10841-006-9058-1

[ece37956-bib-0057] Tscharntke, T., Gathmann, A., & Steffan‐Dewenter, I. (1998). Bioindication using trap‐nesting bees and wasps and their natural enemies: Community structure and interactions. Journal of Applied Ecology, 35(5), 708–719. 10.1046/j.1365-2664.1998.355343.x

[ece37956-bib-0058] Veen, P., Jefferson, R., De Smidt, J., & Van der Straaten, J. (2009). Grasslands in Europe: Of high nature value. BRILL.

[ece37956-bib-0059] Wagner, D. L. (2020). Insect declines in the anthropocene. Annual Review of Entomology, 65(1), 457–480. 10.1146/annurev-ento-011019-025151 31610138

[ece37956-bib-0060] Wagner, D. L., Fox, R., Salcido, D. M., & Dyer, L. A. (2021). A window to the world of global insect declines: Moth biodiversity trends are complex and heterogeneous. Proceedings of the National Academy of Sciences, 118(2), e2002549117. 10.1073/pnas.2002549117 PMC7812805 33431565

[ece37956-bib-0061] Wickham, H. (2016). ggplot2: Elegant graphics for data analysis (2nd ed.). Springer International Publishing. 10.1007/978-3-319-24277-4

