# Supplementary material for: Preliminary assessment of cavity‐nesting Hymenopterans in a low‐intensity agricultural landscape in Transylvania
Source: Ecol Evol. 2021 Aug 1;11(17):11903–14. doi: 10.1002/ece3.7956 (PMC8427617; doi:10.1002/ece3.7956)
Supplement: Supplementary file 1 — Supplementary Material [file ECE3-11-11903-s001.docx]

**Supplementary Information to: Preliminary assessment of cavity-nesting Hymenopterans in a low-intensity agricultural landscape in Transylvania**

**Károly Lajos^1^, Imre Demeter^1^, Róbert Mák***^2^***, Adalbert Balog^3^, Miklós Sárospataki^1^**

*^1^Department of Zoology and Ecology, Hungarian University of Agriculture and Life Sciences, Páter Károly Str. 1, 2100, Gödöllő, Hungary.*

*^2^Patak, Arany János Str. 20, 2648, Hungary*

*^3^Department of Horticulture, Faculty of Technical and Human Science, Sapientia Hungarian University of Transylvania, Sighisoara Str. 1C., 540485, Tirgu-Mures, Romania.*

* Correspondence to: [karoly.lajos@hotmail.com](mailto:karoly.lajos@hotmail.com), [adalbert.balog@ms.sapientia.ro](mailto:adalbert.balog@ms.sapientia.ro), [Sarospataki.Miklos@ uni-mate.hu](mailto:Sarospataki.Miklos@szie.hu)


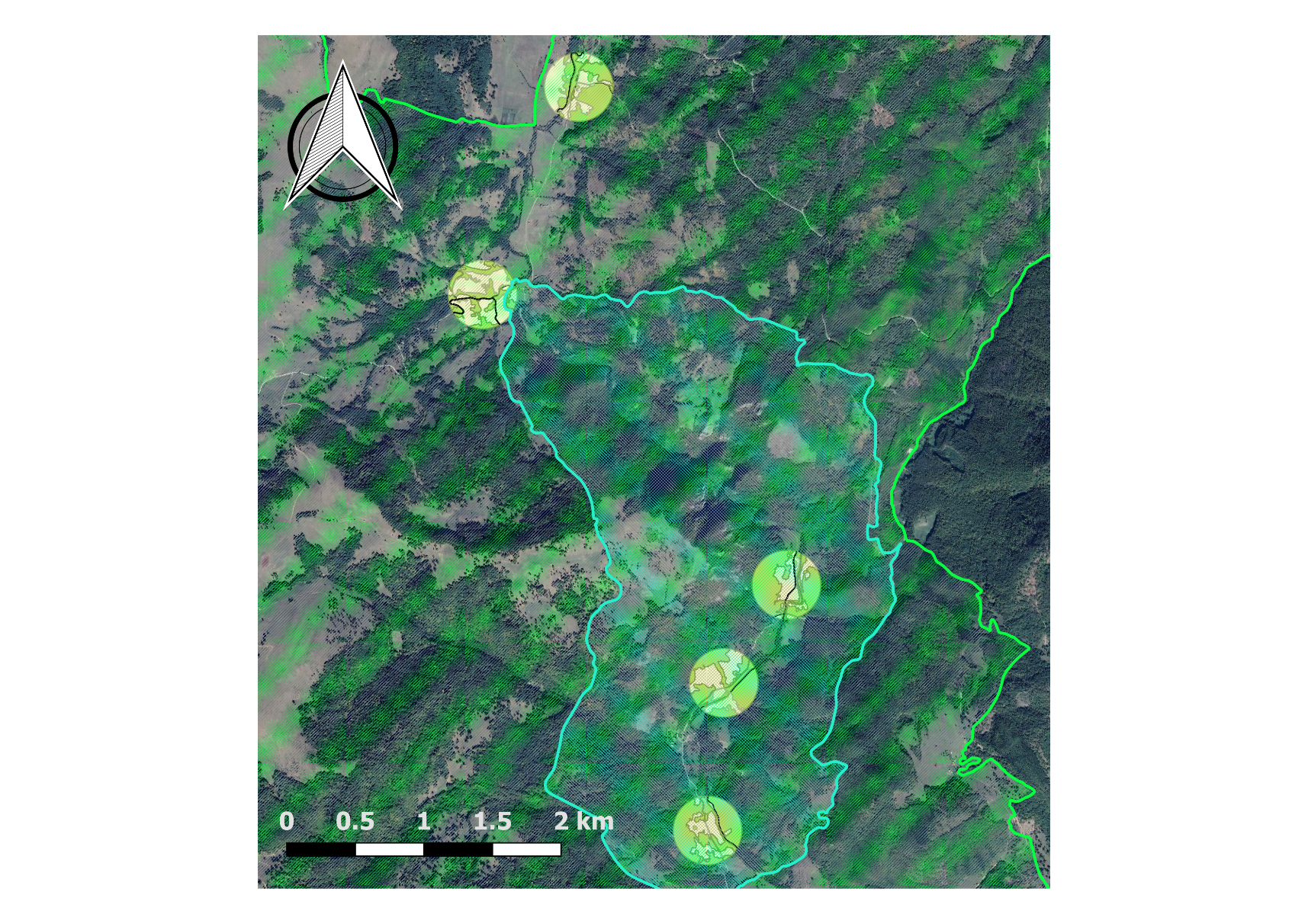


**NV1**

**SV2**

**SV1**

**SV3**

**Vargyas canyon**

**NV2**

**A)**

**B)**


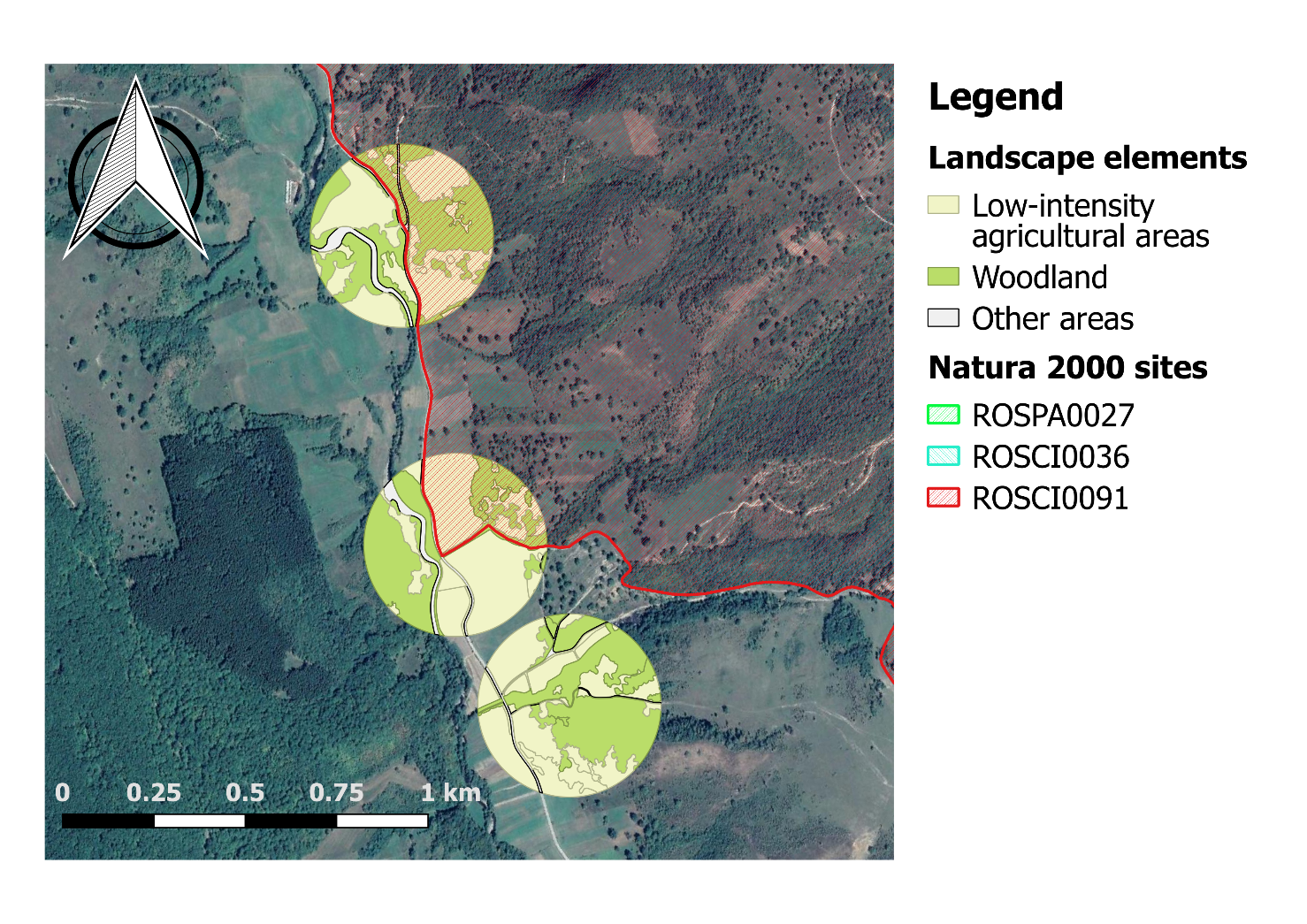


**K3**

**K2**

**K1**

**Figure S1.** **A)** Vector maps of the five study sites located in the Vargyas-valley Northern (NV1-NV2) and Southern (SV1-SV3) to the Vargyas canyon. The base map is a Google Satellite image from 2017. The study sites were situated within the borders of the two Natura 2000 sites ROSPA0027 and ROSCI0036. Note that the Natura 2000 site ROSCI0036 is a part of the site ROSPA0027. **B)** Vector maps of the study sites located in the Körmös-valley (K1-K3). The three study sites were situated at or close to the border of the Natura 2000 site ROSCI0091. The base map is a Google Satellite image from 2019. The vector maps surrounding the study sites were created using the software QGIS 2.18.9 (<http://qgis.osgeo.org>). The vector maps of the Natura 2000 sites were downloaded from the Natura 2000 Network Viewer of the European Environment Agency (https://natura2000.eea.europa.eu/).

**Table S1.** Proportion and edge density of low-intensity agricultural areas within 250 m around the eight study sites. The coordinates of the centre points are given in WGS 84 (EPSG:4326).

| **Site** | **Coordinates (lat/long)** | **Proportion (%)** | **Edge density (m/ha)** |
| --- | --- | --- | --- |
| K1 | 25.6280355 46.17797563 | 48.15 | 345.07 |
| K2 | 25.62770287 46.17029731 | 55.23 | 301.07 |
| K3 | 25.63051194 46.16586504 | 51.83 | 334.23 |
| NV1 | 25.5377679 46.2458034 | 41.70 | 273.93 |
| NV2 | 25.52495344 46.23386036 | 59.49 | 375.99 |
| SV1 | 25.54758536 46.21118958 | 28.35 | 276.63 |
| SV2 | 25.53994534 46.20578119 | 38.96 | 227.54 |
| SV3 | 25.53567693 46.19657022 | 25.91 | 236.91 |

**Table S2.** Spatial autocorrelation (Moran’s I) tested for the total number of occupied brood cells of three cavity-nesting Hymenopteran taxa, the total number of spider specimens from the family of Thomisidae, preyed by the wasp genus *Dipogon* (Dip), and the diversity of the *Trypoxylon* (Try) spider prey. This analysis was conducted in the ETRS89/ETRS-LAEA (EPSG: 3035) coordinate reference system. The number of observations was 8 in each case. Cases of significant spatial autocorrelation are marked bold.

| **Taxon/Group** | **observed** | **expected** | **sd** | **p.value** |
| --- | --- | --- | --- | --- |
| **Hymenopterans** |  |  |  |  |
| *Auplopus* | -0.31 | -0.14 | 0.16 | 0.30 |
| *Megachile* | 0.39 | -0.14 | 0.19 | **0.00** |
| *Osmia* | -0.24 | -0.14 | 0.21 | 0.65 |
| **Spider prey** |  |  |  |  |
| Thomisidae (Dip) | 0.10 | -0.14 | 0.20 | 0.84 |
| Diversity (Try) | 0.11 | -0.14 | 0.20 | 0.19 |

**Table S3.** **A)** Number of reeds and nests, the occupancy of reeds as well as the mean diameters ± SD (in mm) of occupied reed stalks (= nests) per site. **B)** Number of nests, built by seven cavity-nesting Hymenopteran taxa per site. **C)** Number of occupied brood cells per site, found inside these nests. The bottom lines represent the mean values ± SD for all sites.

**A)**

| **Site** | **Reeds** | **Nests** | **Occupancy (%)** | **Diameter ± SD** |
| --- | --- | --- | --- | --- |
| K1 | 641 | 111 | 17.32 | 6.22 ± 1.07 |
| K2 | 564 | 94 | 16.67 | 7.09 ± 0.99 |
| K3 | 575 | 79 | 13.74 | 6.75 ± 1.06 |
| NV1 | 585 | 77 | 13.16 | 6.59 ± 1.17 |
| NV2 | 637 | 140 | 21.98 | 6.27 ± 1.15 |
| SV1 | 601 | 157 | 26.12 | 6.96 ± 0.96 |
| SV2 | 595 | 137 | 23.03 | 6.76 ± 0.92 |
| SV3 | 659 | 195 | 29.59 | 6.29 ± 1.12 |
|  | 607.13 ± 34.43 | 123.75 ± 41.16 | 20.20 ± 5.93 | 6.62 ± 0.33 |

**B)**

| **Site** | **Auplopus** | **Dipogon** | **Eumeninae** | **Hylaeus** | **Megachile** | **Osmia** | **Trypoxylon** |
| --- | --- | --- | --- | --- | --- | --- | --- |
| K1 | 8 | 17 | 51 | 7 | 1 | 4 | 23 |
| K2 | 0 | 8 | 13 | 6 | 4 | 0 | 63 |
| K3 | 1 | 27 | 2 | 14 | 8 | 4 | 23 |
| NV1 | 3 | 27 | 5 | 17 | 2 | 4 | 19 |
| NV2 | 1 | 18 | 18 | 8 | 1 | 2 | 92 |
| SV1 | 2 | 25 | 35 | 6 | 2 | 0 | 87 |
| SV2 | 1 | 22 | 18 | 2 | 0 | 4 | 90 |
| SV3 | 2 | 14 | 10 | 1 | 0 | 5 | 163 |
|  | 2.25 ±  2.49 | 19.75 ±  6.76 | 19.00 ±  16.41 | 7.63 ±  5.48 | 2.25 ±  2.66 | 2.88 ±  1.96 | 70.00 ±  49.12 |

**C)**

| **Site** | **Auplopus** | **Dipogon** | **Eumeninae** | **Hylaeus** | **Megachile** | **Osmia** | **Trypoxylon** |
| --- | --- | --- | --- | --- | --- | --- | --- |
| K1 | 29 | 42 | 151 | 31 | 5 | 13 | 46 |
| K2 | 0 | 14 | 33 | 11 | 20 | 0 | 288 |
| K3 | 8 | 73 | 7 | 32 | 24 | 22 | 82 |
| NV1 | 8 | 62 | 16 | 80 | 2 | 36 | 68 |
| NV2 | 8 | 52 | 46 | 25 | 5 | 4 | 288 |
| SV1 | 17 | 71 | 125 | 28 | 5 | 0 | 371 |
| SV2 | 1 | 55 | 55 | 8 | 0 | 29 | 361 |
| SV3 | 6 | 53 | 48 | 7 | 0 | 37 | 611 |
|  | 9.63 ±  9.40 | 52.75 ±  18.71 | 60.13 ±  51.19 | 27.75 ±  23.51 | 7.63 ±  9.18 | 17.63 ±  15.54 | 264.38 ±  193.27 |

**Table S4. A)** Results of linear models (LMs) testing for the relationships between the number of nests and occupied brood cells for seven cavity-nesting Hymenopteran taxa. The number of observations was 8 in each case. **B)** Mean number of occupied brood cells per nest and site. The bottom line represents the mean values ± SD for all sites.

**A)**

| **Taxon** | **Estimate** | **Std. Error** | **t value** | **Pr(>\|t\|)** |
| --- | --- | --- | --- | --- |
| *Auplopus* | 3.35 | 0.71 | 4.76 | 0.00 |
| *Dipogon* | 2.50 | 0.49 | 5.16 | 0.00 |
| Eumeninae | 3.06 | 0.26 | 11.89 | 0.00 |
| *Hylaeus* | 3.76 | 0.85 | 4.45 | 0.00 |
| *Megachile* | 3.21 | 0.52 | 6.15 | 0.00 |
| *Osmia* | 6.98 | 1.54 | 4.55 | 0.00 |
| *Trypoxylon* | 3.86 | 0.31 | 12.35 | 0.00 |

**B)**

| **Site** | **Auplopus** | **Dipogon** | **Eumeninae** | **Hylaeus** | **Megachile** | **Osmia** | **Trypoxylon** |
| --- | --- | --- | --- | --- | --- | --- | --- |
| K1 | 3.63 | 2.47 | 2.96 | 4.43 | 5.00 | 3.25 | 2.00 |
| K2 | 0.00 | 1.75 | 2.54 | 1.83 | 5.00 | 0.00 | 4.57 |
| K3 | 8.00 | 2.70 | 3.50 | 2.29 | 3.00 | 5.50 | 3.57 |
| NV1 | 2.67 | 2.30 | 3.20 | 4.71 | 1.00 | 9.00 | 3.58 |
| NV2 | 8.00 | 2.89 | 2.56 | 3.13 | 5.00 | 2.00 | 3.13 |
| SV1 | 8.50 | 2.84 | 3.57 | 4.67 | 2.50 | 0.00 | 4.26 |
| SV2 | 1.00 | 2.50 | 3.06 | 4.00 | 0.00 | 7.25 | 4.01 |
| SV3 | 3.00 | 3.79 | 4.80 | 7.00 | 0.00 | 7.40 | 3.75 |
|  | 4.35 ±  3.36 | 2.65 ±  0.58 | 3.27 ±  0.72 | 4.01 ±  1.63 | 2.69 ±  2.19 | 4.30 ±  3.49 | 3.61 ±  0.79 |

**Table S5.** Number of spider specimens from different families per site, preyed by the solitary wasp genera **A)** *Trypoxylon* and **B)** *Dipogon*. The bottom line represents the mean values ± SD for all sites.

**A)**

| **Site** | **Araneidae** | **Linyphiidae** | **Salticidae** | **Tetragnathidae** | **Theridiidae** | **Thomisidae** | **Trachelidae** |
| --- | --- | --- | --- | --- | --- | --- | --- |
| K1 | 6 | 0 | 0 | 0 | 15 | 0 | 0 |
| K2 | 120 | 75 | 1 | 19 | 52 | 6 | 1 |
| K3 | 30 | 7 | 0 | 2 | 6 | 0 | 0 |
| NV1 | 3 | 14 | 0 | 0 | 5 | 0 | 0 |
| NV2 | 31 | 36 | 1 | 7 | 0 | 0 | 0 |
| SV1 | 333 | 17 | 2 | 0 | 4 | 4 | 0 |
| SV2 | 203 | 8 | 0 | 3 | 43 | 0 | 0 |
| SV3 | 388 | 18 | 0 | 0 | 3 | 0 | 0 |
|  | 139.25 ±  152.98 | 21.88 ±  23.95 | 0.50 ±  0.76 | 3.88 ±  6.58 | 16.00 ±  20.06 | 1.25 ±  2.38 | 0.13 ±  0.35 |

**B)**

| **Site** | **Araneidae** | **Linyphiidae** | **Salticidae** | **Theridiidae** | **Thomisidae** | **Trachelidae** |
| --- | --- | --- | --- | --- | --- | --- |
| K1 | 0 | 0 | 0 | 0 | 20 | 0 |
| K2 | 0 | 0 | 0 | 0 | 0 | 0 |
| K3 | 0 | 0 | 0 | 0 | 1 | 0 |
| NV1 | 0 | 0 | 0 | 0 | 17 | 0 |
| NV2 | 0 | 0 | 0 | 0 | 8 | 0 |
| SV1 | 0 | 1 | 1 | 1 | 26 | 0 |
| SV2 | 1 | 0 | 1 | 0 | 7 | 1 |
| SV3 | 0 | 0 | 0 | 0 | 14 | 0 |
|  | 0.13 ±  0.35 | 0.13 ±  0.35 | 0.25 ±  0.46 | 0.13 ±  0.35 | 11.63 ±  9.21 | 0.13 ±  0.35 |
